# Thalamic Atrophy Contributes to Low Slow Wave Sleep in Neuromyelitis Optica Spectrum Disorder

**DOI:** 10.14336/AD.2016.0419

**Published:** 2016-12-01

**Authors:** Lei Su, Yujuan Han, Rong Xue, Kristofer Wood, Fu-Dong Shi, Yaou Liu, Ying Fu

**Affiliations:** ^1^Department of Neurology, Tianjin Neurological Institute, Tianjin Medical University General Hospital, Tianjin 300052, China; ^2^Department of Radiology, Tianjin Third Central Hospital, Tianjin, 300170, China; ^3^Department of Neurology, Barrow Neurological Institute, St. Joseph’s Hospital and Medical Center, Phoenix, AZ 85013, USA; ^4^Department of Radiology and Nuclear Medicine, Neuroscience Campus Amsterdam, VU University Medical Center, Amsterdam, Amsterdam 1007 MB, The Netherlands; ^5^Department of Radiology, Xuanwu Hospital, Capital Medical University, Beijing 100053, China

**Keywords:** neuromyelitis optica spectrum disorder, slow wave sleep, brain structure, magnetic resonance imaging

## Abstract

Slow wave sleep abnormality has been reported in neuromyelitis optica spectrum disorder (NMOSD), but mechanism for such abnormality is unknown. To determine the structural defects in the brain that account for the decrease of slow wave sleep in NMOSD patients. Thirty-three NMOSD patients and 18 matched healthy controls (HC) were enrolled. Polysomnography was used to monitor slow wave sleep and three-dimensional T1-weighted MRIs were obtained to assess the alterations of grey matter volume. The percentage of deep slow wave sleep decreased in 93% NMOSD patients. Compared to HC, a reduction of grey matter volume was found in the bilateral thalamus of patients with a lower percentage of slow wave sleep (FWE corrected at cluster-level, p < 0.05, cluster size > 400 voxels). Furthermore, the right thalamic fraction was positively correlated with the decrease in the percentage of slow wave sleep in NMOSD patients (p < 0.05, FDR corrected, cluster size > 200 voxels). Our study identified that thalamic atrophy is associated with the decrease of slow wave sleep in NMOSD patients. Further studies should evaluate whether neurotransmitters or hormones which stem from thalamus are involved in the decrease of slow wave sleep.

Neuromyelitis optica spectrum disorder (NMOSD) is an autoimmune, inflammatory and demyelinating condition of the central nervous system that is recognized as an anti-aquaporin (AQP)-4 antibody-mediated astro-cytopathy [1]. It can be distinguished from multiple sclerosis (MS) by both clinical and pathological features [2]. Sleep abnormalities in MS have been demonstrated for a long time by significant studies [3]. A recent cross-sectional polysomnographic study found sleep abnormalities in 74% of consecutively enrolled MS patients (49 out of 66 patients) [4]. The core abnormalities in MS are insomnia, restless legs syndrome, periodic limb movement disorders, and sleep-related breathing disorders [5]. Similar to findings in MS, sleep abnormality in NMOSD has also been reported by a recent trial showing decreases in sleep efficiency, slow wave sleep (SWS), arousal index, O_2_ saturation and increases in rapid-eye-movement (REM) sleep, and periodic leg movement (PLM) [6].

Alteration of SWS is the most significant finding in NMOSD. SWS, which is characterized by prominent, high-amplitude, slow wave activity (0.5-2Hz) [7], is considered to be a vital part of non-rapid-eye-movement (NREM) sleep. The aberrance of NREM sleep, as reflected by SWS, may signify synaptic alterations in neurons [8]. Furthermore, accumulating evidence has revealed SWS abnormalities are associated with impaired cognitive performance in patients with neuropsychiatric diseases [9, 10].

Therefore, it is essential to investigate the underlying brain structural defects for such SWS abnormality, which may help identify the mechanism in sleep abnormalities and improve our understanding of the relationship of sleep abnormality and brain impairment in NMOSD.

To achieve this aim, the current study compared grey matter volume (GMV) between NMOSD patients and healthy controls (HC) using voxel-based morphometry (VBM) and region of interest (ROI) methods, and correlated GMV alterations with SWS.

## MATERIALS AND METHODS

### Patient information

Data used for analysis in this study were collected during a previous study conducted between August 2013 and May 2014 [6]. Thirty-three eligible NMOSD patients satisfied with Wingerchuck 2007 criteria [1] (30 NMO, 3 longitudinally extensive transverse myelitis) from Tianjin Medical University General Hospital were enrolled, which were in stable state. Twenty-three (69.70%) patients were AQP-4 antibody positive (20 in 30 NMO, 3 longitudinally extensive transverse myelitis). Serum AQP-4 antibodies was tested in our immune laboratory, using a cell-based assay with quantitative flow cytometry assay as described previously [11, 12]. Eighteen age and gender matched healthy controls with no sleep abnormalities were recruited. The main demographic and clinical characteristics were recorded and all the participants underwent overnight video polysomnography (PSG) examinations (Nicolet v32, Natus Medical Incorporated, Pleasanton, CA). The percentage of SWS was recorded by a specialist. MRI scans were acquired using a 3.0-Tesla MR system (Discovery MR750, General Electric, Milwaukee, WI, USA), included 3D high-resolution T1 as well as conventional T2 and fluid attenuated inversion recovery (FLAIR). Brain Lesions were examined and measured by a neuroradiologist. The volumes of grey matter (GM), white matter (WM), and cerebral spinal fluid (CSF) were calculated using VBM8 on 3D high-resolution T1 images. The anatomical automatic labeling (AAL) brain atlas template was used to extract the volume of region of interesting (ROI).

### MRI protocol

MRI scans were completed immediately after polysomnography (PSG) examination. MRI data were acquired using a 3.0-Tesla MR system (Discovery MR750, General Electric, Milwaukee, WI, USA), using an eight-channel phased array head coil. Tight but comfortable foam padding was used to minimize head motion, and earplugs were used to reduce scanner noise.

Conventional brain MRIs (axial T1, T2 and flair images) were performed to detect brain lesions. Lesion imaging in brain were classified as normal, nonspecific, multiple sclerosis (MS)-like, neuromyelitis optica (NMO)-like, or acute disseminated encephalomyelitis (ADEM)-like lesions by an experienced neuraradiologist. Imaging characteristics were classified as: “MS-like” which were seen in regions considered typical of MS (ie, periventricular, juxtacortical, callosal, and infratentorial), “nonspecific” which described a number of small white matter lesions with no MS features. “NMO-like” which were lesions surrounding the fourth ventricle, hypothalamus or aqueduct lesions as previously described and “ADEM-like“ which were lesions in deep grey nuclei or fluffy white matter.

Sagittal 3D T1-weighted images were acquired by a brain volume (BRAVO) sequence with the following parameters: repetition time (TR) = 8.2 ms, echo time (TE) = 3.2 ms, flip angle = 12°, field of view = 256 mm × 256 mm; matrix = 256 × 256; slice thickness = 1 mm, no gap, and 188 sagittal slices.

Voxel-based morphometry (VBM) was used to assess grey matter volume (GMV) alteration between healthy controls (HC) and patients with low percentage of slow wave sleep (SWS). The GMV of each voxel was processed using VBM8 toolbox (www.dbm.neuro.uni-jena.de/vbm/), incorporated in SPM8 (www.fil.ion.ucl.ac.uk/spm) running under MATLAB R2012b. T1 images were normalized and segmented into grey matter (GM), white matter (WM), and cerebrospinal fluid (CSF). The GM concentration map was first registered into the Montreal Neurological Institute (MNI) space using an affine transformation, and then the images were non-linearly normalized using the diffeomorphic anatomical registration through the exponentiated Lie algebra (DARTEL) technique and were resampled to a voxel size of 1.5 mm × 1.5 mm × 1.5 mm. The non-linear determinants derived from the previous step were then multiplied by the GM concentration map to yield the GMV of each voxel. Finally, a 6 mm × 6 mm × 6 mm, full width at half maximum (FWHM) Gaussian kernel was used to smooth the images. After spatial preprocessing, the normalized, modulated, and smoothed GMV maps were used for statistical analysis.

### Statistical analysis

The results are expressed as mean ± SD for continuous variables and probability for categorical variables. Inter-group differences were compared descriptively using chi-squared tests or Fisher’s exact test for categorical measures. The Kolmogorov-Smirnov tests were used to assess normality of the variables. For normally distributed variables, differences were assessed using Student’s t-test and associations were assessed with Pearson correlation analysis. For variables that were not normally distributed or non-parametric, Wilcoxon Rank Sum Test was used. Statistical significance was defined as *p* < 0.05. Statistical analysis was performed with GraphPad Prism Version 5 (San Diego, USA).

Statistical analysis of structural image data was conducted as follows: voxel-based independent two-sample *t*-test was performed to identify brain regions with significant differences between groups in GMV between HC and NMOSD with low percentage SWS. Statistical tests were evaluated at a significance level of p < 0.05 corrected for multiple comparisons with the family-wise error rate (FWE) at cluster level and Cluster size must be greater than 400 voxels. Correlation between the percentage of SWS and significant GM regions was detected using voxel-level analysis. Multiple comparisons were corrected using a false discovery rate (FDR) method with a corrected threshold of *p* < 0.05, cluster size > 200 voxels.

## RESULTS

### Demographic and SWS percentage in patients with NMOSD

Five patients with NMOSD were excluded due to MRI image artifacts. The remaining MRI images of 28 patients were included in the study. There was no other missing data with demographic or other measured variables in the patients and healthy controls.

**Table 1:** Characteristics of neuromyelitis optica spectrum disorder patients with low slow wave sleep and healthy controls.

Characteristics	HC, n=18	NMOSD with low SWS, n=26	*P*
Age (y)	48.11 ± 11.33	47.00 ± 14.52	0.7981^$^
Female, NO. (%)	15 (83%)	21 (81%)	1.000^&^
Annual relapse rate	NA	0.85 ± 0.51	-
EDSS score	NA	3.19 ± 2.16	-
Disease duration (y)	NA	4.17 ± 3.06	-
SWS (%)	19.70±10.01	7.50 ± 4.87	< 0.0001^*^
MRI brain classification
Normal, NO. (%)	18 (100%)	12 (46%)	-
Nonspecific, NO. (%)	NA	10 (38%)	-
MS-like, NO. (%)	NA	3 (12%)	-
NMO-like, NO. (%)	NA	0 (0%)	-
ADEM-like, NO. (%)	NA	1 (4%)	-
Grey matter fraction	0.47 ± 0.02	0.46 ± 0.02	0.0667^*^
Right thalamic fraction	0.33 ± 0.03	0.29 ± 0.02	< 0.0001^*^
Left thalamic fraction	0.31 ± 0.03	0.29 ± 0.03	0.0037^*^

NMOSD = neuromyelitis optica spectrum disorder; HC = healthy control; EDSS = expanded disability status scale; SWS = slow wave sleep; MS = multiple sclerosis; NMO = neuromyelitis optica; ADEM =acute disseminated encephalomyelitis; NO. = number; NA= not applicable; Data are mean ± SD. Lesion imaging in brain were classified as MS-like if lesions were seen in regions considered typical of MS (ie, periventricular, juxtacortical, callosal, and infratentorial); the term “nonspecific” means a small number of white matter lesions with no MS features. Lesions were classified as NMO-like when surrounding the fourth ventricle, hypothalamus or aqueduct lesions as previously described, and lesions in deep grey nuclei or fluffy white matter were classified as ADEM-like.

$ Wilcoxon signed rank test;

& Chi-square test;

* Student’s t-test.

The mean and SD for SWS percent in healthy controls was 19.70 (10.01) %. SWS percentage decreased more than 1.96 SDs from the mean percentage of healthy controls were considered to have low SWS sleep and the cut-off value of patient with low SWS sleep was 15% in this study. Two patient’s percentages of SWS were greater than 15%, and these two patients were considered as normal SWS sleep. In order to explore the potential impact factors on decrease of SWS, we excluded the two patients without low SWS sleep. Therefore we only compared patients with low a percentage of SWS (n=26) and matched HC (n=18) in the study. There were no differences in age and gender in the two groups (Table 1).

### MRI manifestation of patients with reduced SWS

MRIs of patients brains were considered abnormal in 14 of the 26 cases and were classified as nonspecific (n = 10), MS-like (n = 3) and ADEM-like (n = 1); no patients had NMO-like lesions (Table 1).

**Figure 1.**
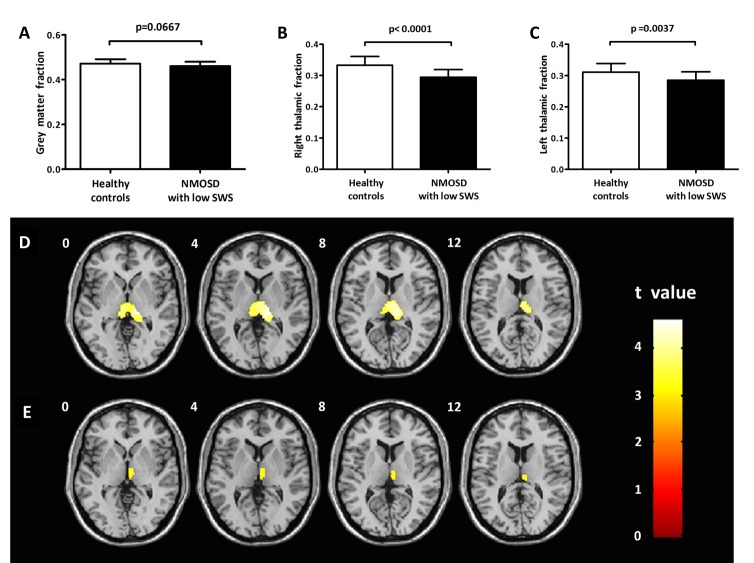
**Association of grey matter volume (GMV) with the percentage of slow wave sleep (SWS) in patients with neuromyelitis optica spectrum disorder (NMOSD).** (**A**) Comparison of the total GMV between healthy controls (HC) and NMOSD patients with low SWS. Comparison of (**B**) right thalamic fraction (TF) and (**C**) left TF between healthy controls (HC) to NMOSD patients with low SWS. (**D**) Comparison of GMV between healthy controls (HC) and NMOSD patients with low SWS based on voxel-level analysis (FWE corrected at cluster-level, *p* < 0.05, cluster size > 400 voxels). (**E**) Correlation between the percentage of SWS and bilateral thalamus volume by voxel-level analysis (FDR corrected, *p* < 0.05, cluster size > 200 voxels). TF were tested by Student’s *t*-test, and Pearson correlation tests were used to examine associations between TF and percentage of SWS. Statistical significance is defined as p<0.05. Bars represent group mean values; standard deviation of the mean was used.

The GMV was applied to normalize the raw volumes of the brain structures. Grey matter fraction (GMF), the normalized GMV, was calculated as follows: GMF = GMV/total intracranial volume (TIV), where TIV= GMV + white matter volume (WMV) + CSF volume. There was no significant difference with regard to the GMF between patients with a low percentage of SWS and HC (0.46 ± 0.02 vs. 0.47 ± 0.02, *p* = 0.0667) (Table 1) (Fig. 1A).

Comparing patients with a low percentage SWS and HC using VBM, a difference was found in the bilateral thalamus (peak t value = 4.61, cluster size = 1879, p = 0.002, FWE corrected at cluster-level) (Fig. 1D). Therefore, the bilateral thalamus was defined as an ROI. Right thalamus volumes were positively correlated with the percentage of SWS using voxel-level analysis (*p* < 0.05, FDR corrected, cluster size > 200 voxels) (Fig. 1E). We were unable to identify such an association with the percentage of SWS in the left TF. And then the bilateral thalamus volume was extracted using the AAL brain atlas template. Raw thalamic volumes were normalized within each subject by calculating the ratio of the thalamic volume to the intracranial volume. The resulting normalized thalamic volume was referred to as the thalamic fraction (TF). The NMOSD patients with a low percentage of SWS were found to have a significantly lower TF than HC in both the right (0.29 ± 0.02 vs. 0.33 ± 0.03; *p* < 0.0001) (Table1) (Fig. 1B) and the left (0.29 ± 0.03 vs. 0.31 ± 0.03; *p* = 0.0037) (Table1) (Fig. 1C) thalamus.

## DISCUSSION

In this study, our results demonstrated that 93% of patients with NMOSD had reduced SWS, indicating that SWS reduction was highly prevalent in NMOSD patients. Compared with HC, patients with low SWS sleep showed obviously decreased thalamic volume, but not in the whole brain GMV. Furthermore, the right thalamic volume was positively correlated with percentage of SWS in NMOSD patients.

Thalamic atrophy, rather than cerebral atrophy, was identified in NMOSD patients with a low percentage of SWS, suggesting that thalamic atrophy appears to be a key factor in the reduction of SWS in NMOSD. It has previously been reported that the thalamus has a critical role in SWS generation and is intimately involved in the bioelectric and behavioral events that occur during SWS [13]. Several factors possibly contribute to thalamic atrophy in NMOSD. The thalamus has rich reciprocal connectivity with a number of regions in the brain and might be particularly vulnerable to hypometabolism and wallerian degeneration due to demyelination and axonal loss in cerebral white matter [14]. The thalamus is located around the lateral ventricles, where high AQP4 expression has been identified [15], making it more accessible to AQP4-IgG attack. Furthermore, the thalamic atrophy might be a consequence of WM abnormalities, which can already be visualized in a substantial proportion of patients with NMO [16]. Moreover, microstructure injury was found to be widespread in normal appearing white matter in NMO patients [17]. Therefore, further studies would be useful to clarify the potential mechanism. The structural abnormalities we found only in the right thalamus on VBM analysis may be due to the highly stringent statistical threshold (FDR < 0.05) employed in a small number of patients. This notion was supported by automatic thalamic segmentation which showed a reduced volume of both left and right thalami in patients with low percentage of SWS as compared with healthy controls.

Although some pathological studies showed cortical degeneration and meningeal inflammation in NMOSD [18], cortical atrophy was not identified in NMOSD patients with a low percentage of SWS in the current study. This finding is consistent with some previous studies [19], but discordant with others [20]. This discordance may be due to the different analytical methods used or heterogeneous patient groups. The cortical alterations may result from the influence of cytokines produced in both focal and global neuroinflammation [21], which can affect the stability of synaptic connections, thereby contributing to sleep deficits. Therefore, alteration of the cerebral cortex may be confined to the molecular level and unable to be sufficiently studied by structural MRI.

In conclusion, our study identified that thalamic atrophy was associated with decrease of SWS in NMOSD patients, suggesting that thalamic atrophy maybe a potential mechanism for SWS abnormality in NMOSD. However, the number of subjects in this study is not sufficient to allow multivariate analyses to produce more accurate and general conclusions, so, more careful evaluation in large number of subjects will be required in future.

## References

[1] Wingerchuk DM, Lennon VA, Lucchinetti CF, Pittock SJ, Weinshenker BG (2007). The spectrum of neuromyelitis optica. Lancet Neurol, 6: 805-815. 17706564 10.1016/S1474-4422(07)70216-8

[2] Matthews L, Marasco R, Jenkinson M, Kuker W, Luppe S, Leite MI, et al (2013). Distinction of seropositive NMO spectrum disorder and MS brain lesion distribution. Neurology, 80: 1330-1337. 23486868 10.1212/WNL.0b013e3182887957 PMC3656462

[3] Poirier G, Montplaisir J, Dumont M, Duquette P, Decary F, Pleines J, et al (1987). Clinical and sleep laboratory study of narcoleptic symptoms in multiple sclerosis. Neurology, 37: 693-695. 3494212 10.1212/wnl.37.4.693

[4] Veauthier C, Radbruch H, Gaede G, Pfueller CF, Dorr J, Bellmann-Strobl J, et al (2011). Fatigue in multiple sclerosis is closely related to sleep disorders: a polysomnographic cross-sectional study. Mult Scler, 17: 613-622. 21278050 10.1177/1352458510393772

[5] Veauthier C (2015). Sleep disorders in multiple sclerosis. Review. Curr Neurol Neurosci Rep, 15: 21. 25773000 10.1007/s11910-015-0546-0

[6] Song Y, Pan L, Fu Y, Sun N, Li YJ, Cai H, et al (2015). Sleep abnormality in neuromyelitis optica spectrum disorder. Neurol Neuroimmunol Neuroinflamm, 2: e94. 25918736 10.1212/NXI.0000000000000094 PMC4405292

[7] Iber C A-IS, Chesson AL, Quan SF for the American Academy of Sleep Medicine, editors. The AASM Manual for the Scoring of Sleep and Associated Events: Rules, Terminology, and Technical Specifications. Illinois: American Academy of Sleep Medicine; 2007

[8] Huber R, Ghilardi MF, Massimini M, Tononi G (2004). Local sleep and learning. Nature, 430: 78-81. 15184907 10.1038/nature02663

[9] Goder R, Boigs M, Braun S, Friege L, Fritzer G, Aldenhoff JB, et al (2004). Impairment of visuospatial memory is associated with decreased slow wave sleep in schizophrenia. J Psychiatr Res, 38: 591-599. 15458855 10.1016/j.jpsychires.2004.04.005

[10] Hot P, Rauchs G, Bertran F, Denise P, Desgranges B, Clochon P, et al (2011). Changes in sleep theta rhythm are related to episodic memory impairment in early Alzheimer's disease. Biol Psychol, 87: 334-339. 21514358 10.1016/j.biopsycho.2011.04.002

[11] Yang CS, Yang L, Li T, Zhang DQ, Jin WN, Li MS, et al (2013). Responsiveness to reduced dosage of rituximab in Chinese patients with neuromyelitis optica. Neurology, 81: 710-713. 23884041 10.1212/WNL.0b013e3182a1aac7 PMC3776460

[12] Li YJ, Zhang F, Qi Y, Chang GQ, Fu Y, Su L, et al (2015). Association of circulating follicular helper T cells with disease course of NMO spectrum disorders. J Neuroimmunol, 278: 239-246. 25468778 10.1016/j.jneuroim.2014.11.011

[13] de Andres I, Garzon M, Reinoso-Suarez F (2011). Functional Anatomy of Non-REM Sleep. Front Neurol, 2: 70. 22110467 10.3389/fneur.2011.00070 PMC3215999

[14] Houtchens MK, Benedict RH, Killiany R, Sharma J, Jaisani Z, Singh B, et al (2007). Thalamic atrophy and cognition in multiple sclerosis. Neurology, 69: 1213-1223. 17875909 10.1212/01.wnl.0000276992.17011.b5

[15] Pittock SJ, Weinshenker BG, Lucchinetti CF, Wingerchuk DM, Corboy JR, Lennon VA (2006). Neuromyelitis optica brain lesions localized at sites of high aquaporin 4 expression. Arch Neurol, 63: 964-968. 16831965 10.1001/archneur.63.7.964

[16] Kim HJ, Paul F, Lana-Peixoto MA, Tenembaum S, Asgari N, Palace J, et al (2015). MRI characteristics of neuromyelitis optica spectrum disorder: an international update. Neurology, 84: 1165-1173. 25695963 10.1212/WNL.0000000000001367 PMC4371410

[17] Liu Y, Duan Y, He Y, Yu C, Wang J, Huang J, et al (2012). A tract-based diffusion study of cerebral white matter in neuromyelitis optica reveals widespread pathological alterations. Mult Scler, 18: 1013-1021. 22183932 10.1177/1352458511431731

[18] Saji E, Arakawa M, Yanagawa K, Toyoshima Y, Yokoseki A, Okamoto K, et al (2013). Cognitive impairment and cortical degeneration in neuromyelitis optica. Ann Neurol, 73: 65-76. 23378324 10.1002/ana.23721

[19] Sinnecker T, Dorr J, Pfueller CF, Harms L, Ruprecht K, Jarius S, et al (2012). Distinct lesion morphology at 7-T MRI differentiates neuromyelitis optica from multiple sclerosis. Neurology, 79: 708-714. 22855861 10.1212/WNL.0b013e3182648bc8

[20] Sanchez-Catasus CA, Cabrera-Gomez J, Almaguer Melian W, Giroud Benitez JL, Rodriguez Rojas R, Bayard JB, et al (2013). Brain Tissue Volumes and Perfusion Change with the Number of Optic Neuritis Attacks in Relapsing Neuromyelitis Optica: A Voxel-Based Correlation Study. PLoS One, 8: e66271. 23824339 10.1371/journal.pone.0066271 PMC3688888

[21] Yang G, Parkhurst CN, Hayes S, Gan WB (2013). Peripheral elevation of TNF-alpha leads to early synaptic abnormalities in the mouse somatosensory cortex in experimental autoimmune encephalomyelitis. Proc Natl Acad Sci U S A, 110: 10306-10311. 23733958 10.1073/pnas.1222895110 PMC3690863

